# F85. THE RELATIONSHIP BETWEEN JUMPING TO CONCLUSIONS AND NEUROPSYCHOLOGICAL FUNCTIONING IN SCHIZOPHRENIA

**DOI:** 10.1093/schbul/sby017.616

**Published:** 2018-04-01

**Authors:** Martyna Krężołek, Renata Pionke, Beata Banaszak, Andrzej Kokoszka, Łukasz Gawęda

**Affiliations:** 1 Medical University of Warsaw; 2 Institute of Psychology, Polish Academy of Sciences; 3 Bielański Hospital; 4 Medical University of Warsaw, University Medical Center Hamburg Eppendorf

## Abstract

**Background:**

Patients with schizophrenia display a tendency to jump to conclusions (JTC), although the cognitive mechanisms of JTC remain unknown. The main aim of our study was to investigate the relationship between the subjective and objective measure of JTC and neuropsychological functioning in a sample of people with schizophrenia.

**Methods:**

A total of 85 patients diagnosed (44 females, mean age 42.20, SD=11.42) with schizophrenia were assessed with neuropsychological tests, including executive functions, verbal memory, working memory, processing speed and attention. JTC was assessed with the Fish Task (probability 80:20 and 60:40) and a self-report scale (DACOBS). Symptom severity was assessed with the PANSS. The relationship between JTC and neuropsychological functioning was investigated with correlation and regression analyses.

**Results:**

JTC measured by the 60:40 Fish Task version showed a greater number of moderate associations with neuropsychological variables as compared with the task’s 80:20 version. Self-reported JTC turned out to be negatively correlated with CVLT and the Backward Digit Span. The regression analyses model, when controlling for duration of illness, age and symptoms, showed that neuropsychological variables associated with the tests, i.e. CVLT and Forward Digit Span, were specifically related to JTC measured by Fish Task 60:40. These variables turned out to be insignificant predictors of JTC 80:20 and JTC as the DACOBS subscale. JTC measured using Fish Task (60:40) was correlated only with disorganization.

**Discussion:**

The results from the present study suggest that the relationship between decision making during the reasoning task and neuropsychological functioning is modulated by task demands. In particular, verbal working memory deficits are implicated in more hasty decision making when the task is demanding.

